# Positive Selection by Purified MHC Class II / Thymic Epithelial Cells *In Vitro*: Costimulatory Signals Mediated by B7 Are Not Involved

**DOI:** 10.1155/1994/75434

**Published:** 1994

**Authors:** Eric J. Jenkinson, Graham Anderson, Nel C. Moore, Christopher A. Smith, John J. T. Owen

**Affiliations:** 1 Department of Anatomy The Medical School University of Birmingham Birmingham B15 2TT UK; 2 Department of Biological Sciences Keele University Keele Staffordshire ST5 5BG UK

**Keywords:** Positive selection, organ culture, costimulation, fetal thymus, thymic epithelium

## Abstract

We have investigated the possibility that the costimulatory signals required for activation of mature T cells also play a role in providing differentiation signals for positive selection during T-cell development. We show that purified MHC Class II^+^ thymic epithelial cells are able to support positive selection *in vitro* but lack both the functional capacity to deliver costimulatory signals and expression of the costimulatory ligand B7. Our results suggest that the additional signals provided by costimulatory ligands are not required for TCR-mediated positive selection, although other ancillary signals provided by thymic epithelial cells may be involved.


*Developmental Immunology*, 1994, Vol 3, pp. 265–271
Reprints available directly from the publisher
Photocopying permitted by license only

© 1994 Harwood Academic Publishers GmbH
Printed in Singapore

### Positive Selection by Purified MHC Class II$^+$ Thymic Epithelial Cells *In Vitro*: Costimulatory Signals Mediated by B7 Are Not Involved

ERIC J. JENKINSON,*† GRAHAM ANDERSON,† NEL C. MOORE,† CHRISTOPHER A. SMITH,‡ and JOHN J.T. OWEN†

†*Department of Anatomy, The Medical School, University of Birmingham, Birmingham B15 2TT, U.K.*
‡*Department of Biological Sciences, Keele University, Keele, Staffordshire ST5 5BG, U.K.*

We have investigated the possibility that the costimulatory signals required for activation of mature T cells also play a role in providing differentiation signals for positive selection during T-cell development. We show that purified MHC Class II$^+$ thymic epithelial cells are able to support positive selection *in vitro* but lack both the functional capacity to deliver costimulatory signals and expression of the costimulatory ligand B7. Our results suggest that the additional signals provided by costimulatory ligands are not required for TCR-mediated positive selection, although other ancillary signals provided by thymic epithelial cells may be involved.

KEYWORDS: Positive selection, organ culture, costimulation, fetal thymus, thymic epithelium.

### INTRODUCTION

T-cell precursors entering the thymus undergo a program of proliferation and differentiation to produce a population of CD4$^+$8$^+$ cortical thymocytes expressing low levels of the T-cell receptor (TCR). Further maturation into single positive CD4$^+$ or CD8$^+$ cells requires positive selection and is triggered by signaling through the TCR in those cells recognizing major histocompatibility complex (MHC) molecules on the thymic epithelium (reviewed in von Boehmer, 1992). However, engaging the TCR on CD4$^+$8$^+$ cells can also lead to negative selection, by the induction of apoptosis or programmed cell death (Smith et al., 1989). Thus, TCR-mediated signaling can have at least two different consequences in CD4$^+$8$^+$ cells, suggesting that additional factors may be involved in regulating the consequences of TCR engagement at this stage of development.

In mature T cells, activation and proliferation in response to antigen requires dual signaling involving both TCR ligation and costimulatory signals mediated by interactions between CD28 on T cells and B7 on antigen-presenting cells (reviewed in Lui and Linsley, 1992; Jenkins and Johnson, 1993). In contrast, CD28-B7 mediated costimulation does not appear to be required for negative selection (Tan et al., 1992; Jones et al., 1993). However, a possible role for costimulatory signals in positive selection has been raised by the finding of a number of similarities between this process and activation (Bendelac et al., 1992).

Recently, we have developed a reaggregate organ culture system that supports the differentiation of CD4$^+$8$^+$ cells *in vitro* when they are combined with selected thymic stromal cells (Jenkinson et al., 1992). This has enabled us to address directly the role of costimulatory signals in positive selection by examining the ability of thymic epithelium to provide these signals under conditions where it is competent to support positive selection. We show that purified MHC Class II$^+$ epithelial cells can supply all the signals required for positive selection but lack both the ability to provide functional costimulatory signals and expression of the costimulatory ligand B7. These results provide direct evidence that the costimulatory signals involved in the activation of mature T cells are not required for the positive selection and consequent differentiation of CD4$^+$8$^+$ thymocytes.

*Corresponding author.

### RESULTS AND DISCUSSION

### Purified MHC Class II$^+$ Epithelial Cells Support Positive Selection Resulting in Phenotypic and Functional Maturation of CD4$^+$8$^+$ Thymocytes *In Vitro*

We have previously shown that dGuo treated fetal thymus lobes consist of a mixture of cortical and medullary epithelial cells, fibroblasts, and macrophages, and contain all the components required to support the maturation of CD4$^+$8$^+$ thymocytes into functionally competent single positive CD4$^+$ or CD8$^+$ cells (Jenkinson et al., 1992). To determine whether purified MHC Class II$^+$ cortical epithelial cells can act as the sole source of signals required for maturation, we looked at their ability to support the generation of TCR$^+$ single positive CD4$^+$ or CD8$^+$ cells from TCR$^-$ CD4$^+$8$^+$ precursors in reaggregate cultures. This approach excludes the involvement of other stromal cell types as source of additional differentiation signals, unlike studies where thymic epithelial cell lines (Hugo et al., 1992; Vukmanovic et al., 1992) or bone marrow-derived cells (Bix and Raulet, 1992) have been shown to mediate positive selection when introduced into the thymus *in vivo* where endogenous stromal components are still present.

As shown in Fig. 1, purified MHC Class II$^+$ epithelial cells support both the phenotypic and functional maturation of CD4$^+$8$^+$ cells, resulting in the appearance of CD4$^+$ or CD8$^+$ single positive cells with upregulated levels of TCR and the ability

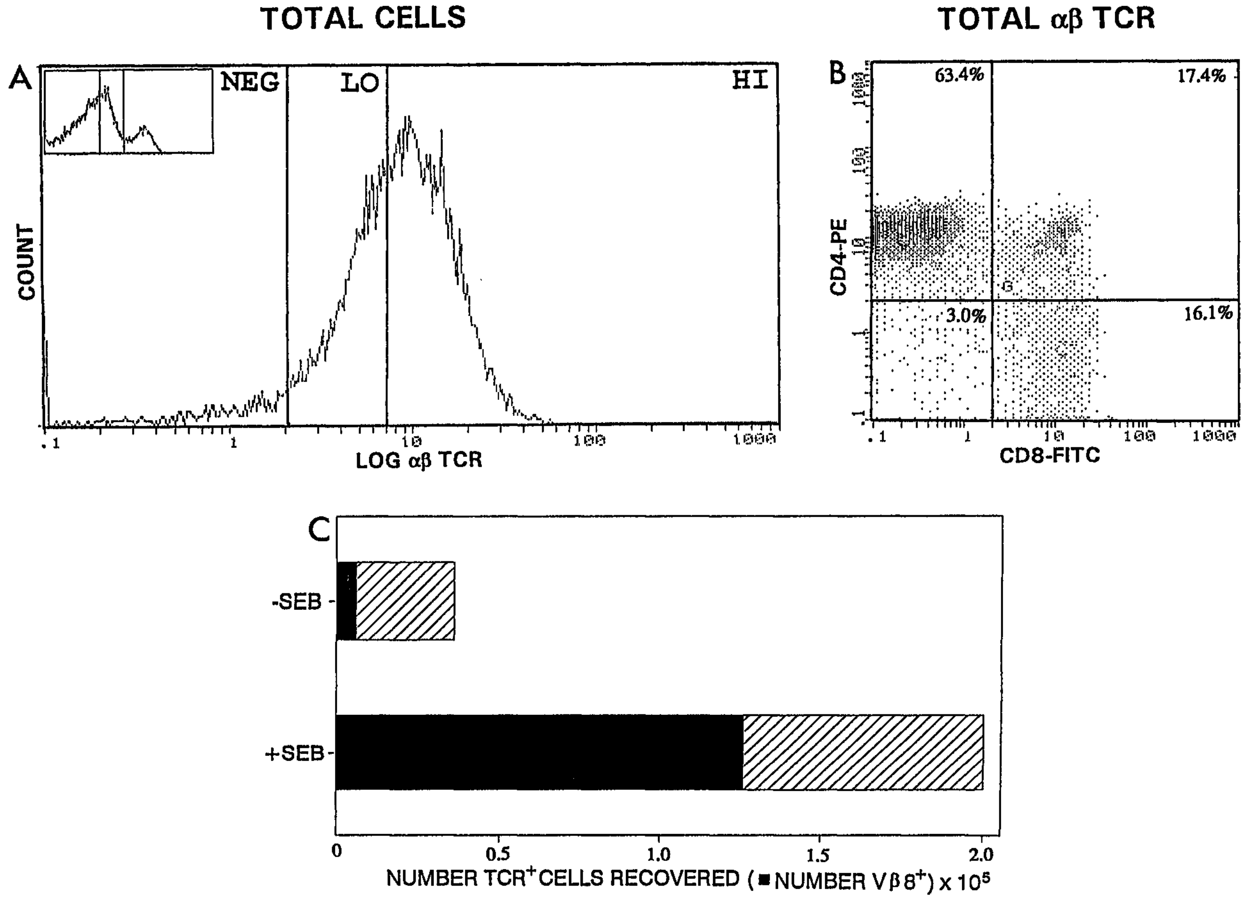

FIGURE 1. Purified MHC Class II$^+$ thymic epithelial cells support positive selection *in vitro*. Three-color flow cytometry analysis shows the phenotype of cells developing in reaggregate cultures of purified epithelium and TCR$^-$CD4$^+$8$^+$ cells. Cultures harvested after 4 days contain about 30% of the original input number of thymocytes. (A) In comparison with normal adult thymocytes (inset), the majority of these cells display upregulated levels of TCR expression and (B) have a single positive CD4$^+$ or CD8$^+$ phenotype, indicating that they have undergone positive selection. (C) This conclusion is further supported by the ability of cells recovered from these cultures to make a specific proliferative response when stimulated with the V$\beta$8 specific superantigen SEB, indicating that functional as well as phenotypic maturation has taken place.

to respond to stimulation by antigen. These maturational changes parallel those occurring as a result of positive selection *in vivo* (reviewed in Hugo et al., 1990; von Boehmer, 1992), indicating the MHC Class II$^+$ thymic epithelial cells, under the conditions of reaggregate cultures, can supply all the signals required for this process.

### Analysis of the Costimulatory Capacity of MHC Class II$^+$ Thymic Epithelial Cells

If costimulatory signals are a requirement for positive selection, the results in the previous section imply that thymic epithelial cells should be able to provide them. Previous attempts to investigate the costimulatory properties of thymic epithelial cells have been based on the ability of monolayer cultures of either epithelial cell lines or thymic nurse-cell preparations to present antigen to mature T cells (Kyewski et al., 1984; Marrack et al., 1989; Lorenz and Allen, 1989). However, none of these preparations has been shown to mediate positive selection and in some cases they actually mediate negative selection (Iwabuchi et al., 1992; Pircher et al., 1993), suggesting either that they are not representative of cells mediating positive selection *in vivo* or that they have lost the capacity to deliver the signals required for this process. This latter possibility is reinforced by the change in surface phenotype seen when epithelial cells are grown in monolayer cultures. (Nonoyama et al., 1989; our unpublished observations).

To investigate the costimulatory function of epithelial cells under conditions where they can mediate positive selection, we examined their ability to stimulate the proliferation of mature (peripheral) V$\beta$8 T cells when presenting the superantigen SEB in reaggregate cultures. Proliferation in response to SEB, like that to conventional antigens, has been shown to require costimulation (Jones et al., 1993). As shown in Fig. 2, no evidence of proliferation, either in terms of increased cell number or of entry of cells into the $G_2$/M phases of the cell cycle, was observed when SEB was presented on thymic epithelial cells in reaggregate cultures. In contrast, marked proliferation in response to SEB was observed in reaggregates of V$\beta8^+$ cells and thymic dendritic cells. This difference was not due to the inability of epithelial cells to bind SEB (Jenkinson et al., 1992) or to differences in the level of Class II antigens available to present SEB on epithelial and dendritic cells (not shown), suggesting that thymic epithelial cells lack the ability of dendritic cells to provide costimulatory signals.

### Thymic Epithelial Cells Mediating Positive Selection Do Not Express the Costimulatory Ligand B7

Recent studies have shown that interactions between CD28 on T cells and B7 on antigen-presenting cells play an important role in providing costimulatory signals for activation (Liu and Linsley, 1992; Jenkins and Johnson, 1993). To determine whether the failure of epithelial cells to provide costimulatory signals was associated with the absence of B7, we examined purified epithelial cells for both B7 mRNA and B7 surface expression. B7 was not detectable in thymic epithelial cells by either technique but was expressed by thymic dendritic cells (Fig. 3) correlating with the differing ability of these two cell types to provide functional costimulation. These findings confirm those recently reported by Jones et al. (1993). In addition, because B7-mediated costimulation has been shown to be a requirement for allograft rejection (Turka et al., 1992), they are consistent with our previous observation that grafts of MHC Class II$^+$ thymic epithelial cells are capable of prolonged survival in immuno-competent histoincompatible recipients (Ready et al., 1984).

Overall, our results suggest that costimulatory signals of the type involved in the activation of mature T cells are not involved in positive selection. However, because TCR signaling alone is insufficient to trigger the maturation of CD4$^+$8$^+$ thymocytes (Swat et al., 1993), it seems likely that other types of costimulatory or additional signals are involved. Our current studies, using reaggregate cultures of CD4$^+$8$^+$ thymocytes and a variety of epithelial and nonepithelial stromal components, suggest that the provision of such signals is uniquely associated with thymic epithelial cells (Anderson et al., in preparation). Identifying these signals should provide an important insight into the control mechanisms for positive selection.

### MATERIALS AND METHODS

### Animals

All material was obtained from Balb/c (H2$^d$) mice. Embryonic material was obtained from timed

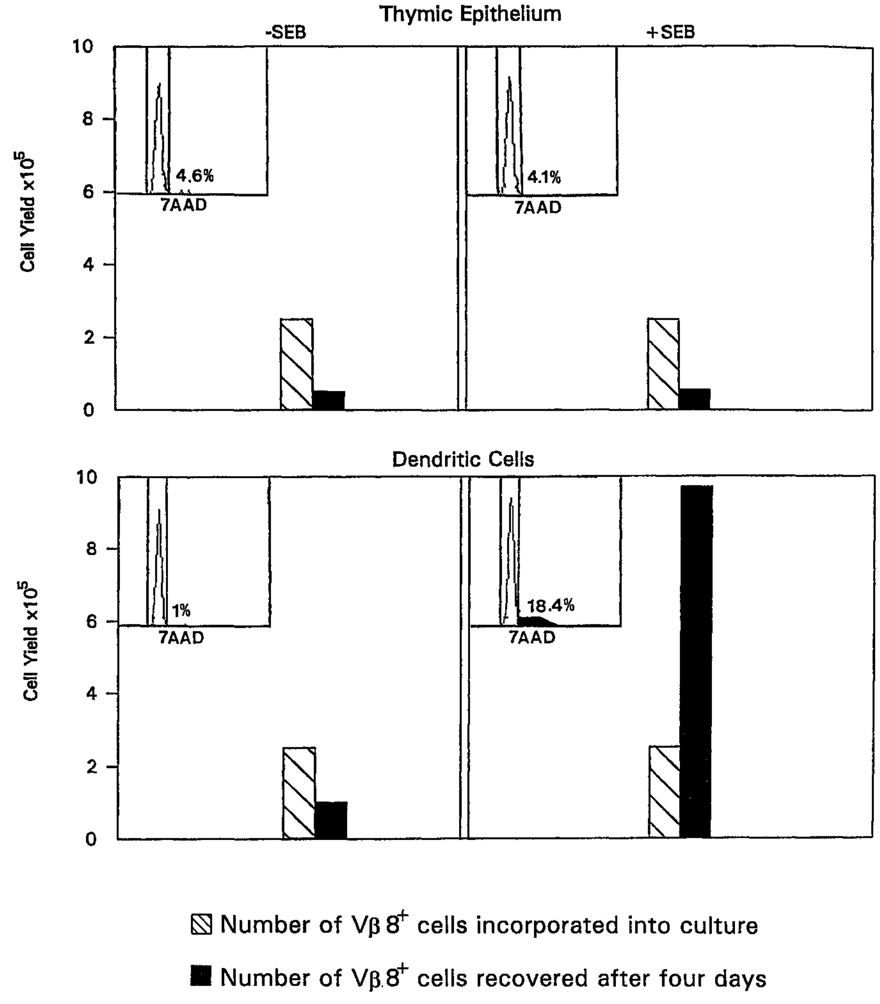

FIGURE 2. Mature V$\beta 8^+$ T cells fail to proliferate in response to SEB presented on MHC Class II$^+$ thymic epithelial cells in reaggregate cultures but respond to SEB presented on thymic dendritic cells. Proliferation in reaggregate cultures was determined by the increase in cell number and by the cell cycle status of recovered cells (insets). Increased cell yields with cells in the $G_2/M$ phase of the cycle were observed only when SEB was presented on dendritic cells. Cultures where SEB was presented on epithelial cells resembled control cultures without SEB and showed no increase in cell number and minimal numbers of cycling cells. Similar results were obtained in three separate experiments.

matings with the day of the vaginal plug designated as day 0.

### Isolation of MHC Class II$^+$ Thymic Epithelial Cells

Purified MHC Class II$^+$ thymic epithelial cells were prepared by immunomagnetic selection from disaggregated fetal thymus lobes as described in detail previously (Jenkinson et al., 1992; Anderson et al., 1993). Briefly, isolated lobes from day 14 fetuses were cultured for 6 days in medium containing dGuo to eliminate lymphoid and dendritic cells, disaggregated and depleted of any residual cells of hemopoietic origin using magnetic beads (Dynal) coated with anti-CD45 (clone M1-9, ATCC). Further depletions were carried out to remove cells expressing the medullary marker A2B5 before MHC Class II$^+$ cells were positively selected on beads coated with anti-Ia$^d$ (clone MK-D6, Becton Dickinson). Beaded cells were collected and washed four times on a magnet to remove unbound cells and the beads removed enzymically by resuspension in 200 $\mu$l of ice-cold Pronase (10 mg/ml, Sigma), followed by a 1–2 min incubation at 37°C. After addition of 1 ml of medium containing 10% FCS, liberated cells and beads were separated on a magnet and the cells washed and collected by centrifugation and subjected to a further round of depletion with A2B5 coated beads. Cells isolated in this way express high levels of MHC Class II together with the cortical epithelium markers ERTR-4 and 4F11E and are free of cells expressing macrophage and fibroblast markers (Anderson et al., 1993).

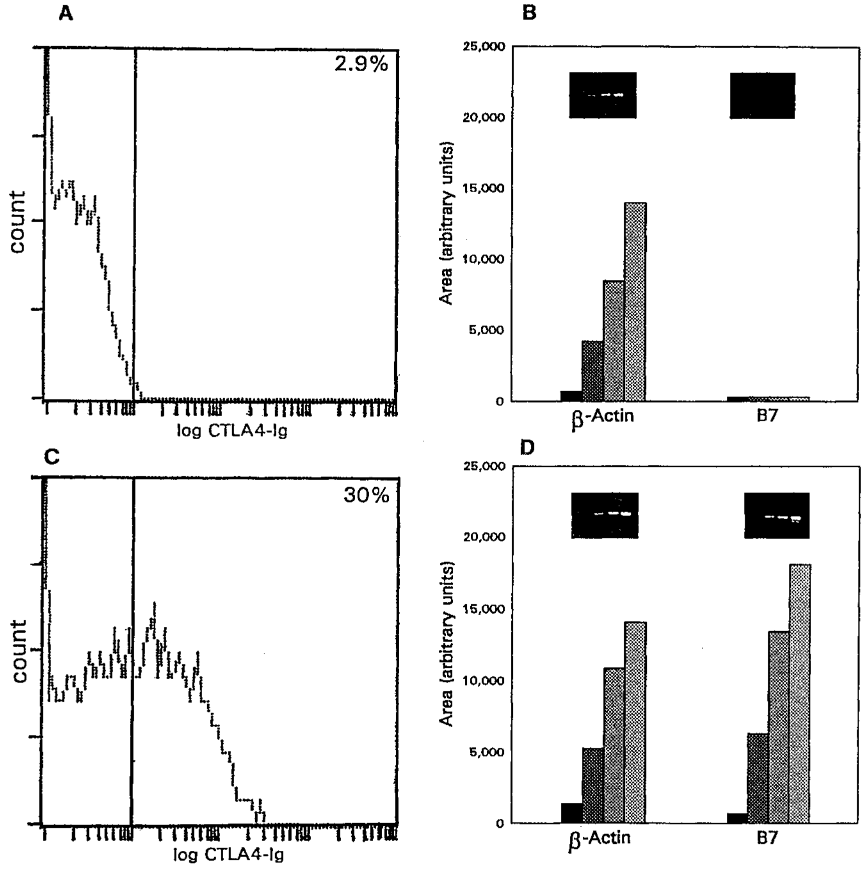

FIGURE 3. B7 expression is not detectable on MHC Class II+ thymic epithelial cells (A, B) but is present on thymic dendritic cells (C, D). B7 surface expression was detected by binding of CTLA-4Ig (A, C) and B7 mRNA by RT-PCR (B, D). FACS profiles were generated by two-color labeling for B7 and MHC Class II. B7 profiles of cells positive for MHC Class II are shown. Background levels (vertical lines) were obtained by omitting CTLA-4Ig from control tubes. Purified cell populations provided the source material for RT-PCR. PCRs were analyzed at cycles 17, 20, 26, and 29 for β-actin and cycles 24, 28, 32, and 36 for B7. Both ethidium bromide-stained gels and data obtained by densitometry are shown. In this experiment, dendritic and epithelial preparations were analyzed in parallel. Similar results were obtained in three separate experiments.

### Preparation of Thymic Dendritic Cells

Thymic dendritic cells were generated from precursors obtained from fetal thymus lobes using a modification of a method described by Inaba et al. (1992) for adult mouse blood. Day 14 fetal thymus lobes were organ cultured for 7 days, teased apart, and the resultant cell suspensions placed in 24-well plates at $1 \times 10^6$ cells/well in medium containing 40 U/ml of recombinant murine GM-CSF (Genzyme). Colonies growing on a monolayer of adherent cells were evident after 1 week and were harvested after 2 to 3 weeks by gentle pipetting. Cells harvested in this way were greater than 90% positive for MHC Class II$^+$ and expressed the dendritic cell markers NLDC-145 and MIDC-8 (Breel et al., 1987).

### Preparation of Reaggregate Cultures

TCR$^-$, CD4$^+$8$^+$/CD4$^-$8$^+$ cells were prepared from suspensions of newborn thymocytes by depletion of CD3$^+$cells followed by positive selection on CD8 using antibody coated magnetic beads as described in detail elsewhere (Jenkinson et al., 1992; Anderson et al., 1993). These cells were mixed with purified MHC Class II$^+$ epithelial cells at a ratio of 2–3:1 and placed as standing drops on nucleopore filters supported on sponge rafts to allow reaggregation into intact thymus lobes. Intact lobes formed within 12 hr and phenotypic maturation, as shown by the appearance of single positive CD4$^+$ or CD8$^+$ cells with high levels of TCR expression (that is, positively selected cells), was determined after 4 days by three-color flow cytometry on cell suspensions liberated by teasing the lobes apart.

Functional maturation of cells recovered from reaggregate cultures was determined by their ability to proliferate in response to stimulation by antigen. Cells were released from reaggregate lobes by gentle mechanical disruption and were mixed with MHC Class II$^+$, B7$^+$ antigen-presenting cells, and cultured for 4 days in the presence or absence of SEB.

Proliferation was assessed by comparing total cell yields and the proportion of SEB specific Vβ8⁺ cells.

### Assessment of Costimulatory Function by Thymic Epithelial Cells

Purified mature $V\beta8^+$ T cells, for use in costimulation assays, were obtained by immunomagnetic selection from cell suspension prepared by mechanical disruption of axial and inguinal lymph nodes from 4–5-week-old Balb/c mice. Liberated suspensions were first depleted of phagocytes, B cells, and dendritic cells by three rounds of depletion with anti-$Ia^d$ coated magnetic beads, followed by positive selection of $V\beta8^+$ cells using beads coated with anti-Vβ8 (clone F23.1) at a ratio of 3:1. Beaded cells were washed four times on a magnet to remove unbound cells and then incubated at 37°C for 2 hr in flat-bottom 96-well plates, during which time most cells shed their beads. Following removal of the beads, liberated cells were collected by centrifugation and counted.

To determine the ability of purified epithelial cells to provide costimulatory signals under conditions where they are known to support the positive selection of developing thymocytes, epithelial cell suspensions were mixed with purified $V\beta8^+$ lymph node T cells at a ratio of 2:1 and allowed to reform reaggregate lobes on nucleopore filters, as described in the previous section. SEB (Sigma) at 10 mg/ml was added to experimental cultures at the outset or omitted in the case of control cultures. For comparison, $V\beta8^+$ cells were also reaggregated with MHC Class $II^+$ thymic dendritic cells in either the presence or absence of SEB. Cultures were harvested after 4 days, mechanically disrupted with fine knives, and counted to determine the total yield of $V\beta8^+$ cells.

As a further measure of proliferation, the cell cycle status of T lymphocytes recovered from reaggregate cultures was assessed using the DNA binding dye 7AAD in conjunction with surface labeling for CD4 and CD8 (Rabinovitch et al., 1986). Cell suspensions were incubated in a mixture of CD4-PE and CD8-FITC (Becton Dickinson), fixed in 80% ethanol, and resuspended in PBS containing 0.1% Tween 20, 0.1 mM EDTA, and 25 μg/ml 7AAD (Sigma) 30 min before analysis. Three-color flow cytometry was carried out on an Elite Dual Laser machine (Coulter) to determine the cycling status of cells expressing either CD4 or CD8. Nonviable cells were excluded by forward and side scatter gating.

### Detection of B7 mRNA by Semiquantitative PCR

Expression of B7 mRNA in either purified thymic MHC Class $II^+$ epithelial cells or purified (MHC Class II selected) thymic dendritic cells was determined using semiquantitative PCR performed as described in detail elsewhere (Moore et al., 1993). In short, cytosolic RNA was extracted from $1\text{–}3 \times 10^5$ cells, treated with DNase I (Pharmacia), and reversed transcribed using M-MLV reverse transcriptase (Gibco-BRL). B7 and β-actin sequences were amplified using one cycle at 94°C for 5 min and 17–50 cycles at 94°C for 30 sec, 50°C for 60 sec, and 72°C for 60 sec. Ten microliters of reaction mix was removed at regular intervals during PCR to encompass the exponential phase of the amplification. PCR fragments, visualized by agarose gel electrophoresis and ethidium bromide staining, were positively identified by size and/or partial DNA sequencing. The intensity of the ethidium bromide stained bands was determined using a gel documentation system (Image Store 5000, UVP, Cambridge, GB) followed by scanning densitometry (E.A.S.Y, UVP). Primer sequences for β-actin have been published before (Moore et al., 1993) and the primer sequences for B7 correspond to bases 474–492 and 1084–1102 of the published murine B7 cDNA sequence (Freeman et al., 1991). The β-actin was used as an internal control for both reverse transcription and the PCR. A negative control incorporating all reagents except cDNA was included in every batch of PCR.

### Detection of B7 Surface Expression by Immunolabeling

Cell-surface expression of B7 on MHC Class II thymic epithelial or thymic dendritic cells was determined by dual labeling, using a mixture of anti-$Ia^d$ (Clone MK-D6, Becton Dickinson) and a B7 binding fusion protein between murine CTLA-4 and human Ig (a kind gift from Dr. P. Lane) followed by anti-human Ig-Biotin (Binding Site, Birmingham, U.K.) and a mixture of anti-mouse Ig-FITC (Caltag) and streptavidin-APC (Becton Dickinson). Analysis was by two-color flow cytometry with side and forward scatter gates set to exclude nonviable cells.

### ACKNOWLEDGMENTS

We are grateful to Alan Murdoch and Deidre McLoughlin for skilled technical support and to Christine Hepherd for preparing the manuscript.

The work was supported by a Programme Grant from the Medical Research Council, U.K. G.A. is the holder of a Wellcome Prize Fellowship.

*(Received July 21, 1993)*

*(Accepted September 23, 1993)*

### REFERENCES

Anderson G., Jenkinson E.J., Moore N.C., and Owen J.J.T. (1993). MHC Class II positive epithelium and mesenchyme cells are both required for T-cell development in the thymus. Nature **362:** 70–73.

Bendelac A., Matzinger P., Sedev R.A., Paul W.E., and Schwartz R.H. (1992). Activation events during thymic selection. J. Exp. Med. **175:** 731–742.

Bix M., and Raulet D. (1992). Inefficient positive selection of T-cells directed by haemopoietic cells. Nature **359:** 330–333.

Breel M., Mebius R.E., and Kraal G. (1987). Dendritic cells of the mouse recognised by two monoclonal antibodies. Eur. J. Immunol. **17:** 1555–1559.

Freeman J.G., Gray G.S., Gimmi C.D., Lombard D.B., Zhov L.J., White M., Fingeroth J.D., Gribbe J.G., and Nadler N.L. (1991). Structure expression and T-cell co-stimulatory activity of the murine homologue of the human B lymphocyte activation antigen B7. J. Exp. Med. **174:** 625–631.

Hugo P., Boyd R.L., Waanders G.A., Petrie, H.T., and Scollay R. (1990). Timing of deletion of auto-reactive $V\beta 86^+$ cells and down-modulation of either CD4 or CD8 on phenotypically distinct $CD4^+8^+$ subsets of thymocytes expressing intermediate or high levels of T cell receptor. Int. Immunol. **3:** 265–272.

Hugo P., Kappler J.W., Godfrey D.I., and Marrach P.C. (1992). A cell line that can induce thymocyte positive selection. Nature **360:** 679–682.

Inaba K., Stainman R.M., Witmer-Pack M., Aya H., Inaba M., Sudo T., Wolpe S., and Schuler G. (1992). Identification of proliferating dendritic cell precursors in mouse blood. J. Exp. Med. **175:** 1157–1167.

Iwabuchi K., Nakayama K.I., McCoy R.L., Wang F., Nishimura T., Habu S., Murphy K.M., and Loh D.Y. (1992). Cellular and peptide requirements for *in vitro* clonal deletion of immature thymocytes. Proc. Natl. Acad. Sci. USA **89:** 9000–9004.

Jenkins M.K., and Johnson J.G. (1993). Molecules involved in T-cell costimulation. Curr. Op. Immunol. **5:** 361–367.

Jenkinson E.J., Anderson G., and Owen J.J.T. (1992). Studies on T-cell maturation on defined thymic stromal cell populations *in vitro*. J. Exp. Med. **176:** 845–853.

Jones L.A., Izon D.J., Nieland J.D., Linsley P.S., and Kruisbeek A.M. (1993). CD28-B7 interactions are not required for intrathymic clonal deletion. Int. Immunol. **5:** 503–512.

Kyewski B.A., Fathman C.G., and Kaplen H.S. (1984). Intrathymic presentation of circulating non-major histocompatibility complex antigens. Nature **308:** 196–199.

Lorenz R.G., and Allen P.M. (1989). Thymus cortical epithelial cells lack full capacity for antigen presentation. Nature **340:** 557–559.

Liu Y., and Linsley P. (1992). Costimulation of T-cell growth. Curr. Op. Immunol. **4:** 265–270.

Marrack P., McCormack J., and Kappler J. (1989). Presentation of antigen, foreign major histocompatibility complex proteins and self by thymic cortical epithelium. Nature **338:** 503–505.

Moore N.C., Anderson G., Smith C.A. Owen J.J.T., and Jenkinson E.J. (1993). Analysis of cytokine gene expression in subpopulations of freshly isolated thymocytes and thymic stromal cells using semi-quantitative polymerase chain reaction. Eur. J. Immunol. **23:** 922–927.

Nonoyama S., Nakayama M., Shiohara T., and Yata J. (1989). Only dull $CD3^+$ thymocytes bind to thymic epithelial cells. The binding is elicited by both CD2/LFA-3 and LFA-1/ICAM-1 interactions. Eur. J. Immunol. **19:** 1631–1635.

Pircher M., Brduscha K., Steinhoff V., Kasai M., Mizvochi T., Zinkernagel R.M., Hengartner H., Kyewski B., and Muller K.P. (1993). Tolerance induction by clonal deletion of $CD4^+8^+$ thymocytes *in vitro* does not require dedicated antigen presenting cells. Eur. J. Immunol. **23:** 669–674.

Rabinovitch P.S., Torres R.M., and Engel D. (1986). Simultaneous cell cycle analysis and two colour surface immunofluorescence using 7-amino-actinomycin-D and single laser excitation: Applications to the study of cell activation and the cell of murine Ly-1 B cells. J. Immunol. **136:** 2769–2775.

Ready A.R., Jenkinson E.J., Kingston R., and Owen J.J.T. (1984). Successful transplantation across major histocompatibility barrier of deoxyguanosine-treated embryonic thymus expressing Class II antigens. Nature **310:** 231–233.

Smith C.A., Williams G.T., Kingston R., Jenkinson E.J., and Owen J.J.T. (1989). Antibodies to the CD3/T-cell receptor complex induce apoptosis in immature thymic culture. Nature **337:** 181–183.

Swat W., Dessing M., von Boehmer H., and Kisielow P. (1993). CD69 expression during selection and maturation of $CD4^+8^+$ thymocytes. Eur J. Immunol. **23:** 739–746.

Tan R., Teh S.J., Ledbetter J.A., Linsley P., and Teh H.S. (1992). B7 costimulates proliferation of $CD4^-8^+$ lymphocytes but is not required for the deletion of immature $CD4^+8^+$ thymocytes. J. Immunol. **149:** 3217–3224.

Turka L.A., Linsley P.S., Liu H., Brady W., Leiden J.M., Wei R.Q., Gibson M., Zheng X.G., Myradal S., Gordon D., et al. (1992). T-cell activation by the CD28 ligand B7 is required for cardiac allograft rejection *in vivo*. Proc. Natl. Acad. Sci. USA **89:** 11102–11106.

von Boehmer H. (1992). Thymic selection: A matter of life and death. Immunol. Today **13:** 454–458.

Vukmanovic S., Grandea A.G., Faas S.J., Knowles B.B., and Bevan M.J. (1992). Positive selective of T-lymphocytes induced by intrathymic injection of a thymic epithelial cell line. Nature **359:** 729–732.

